# Clinical, technical, and implementation characteristics of real-world health applications using FHIR

**DOI:** 10.1093/jamiaopen/ooac077

**Published:** 2022-10-12

**Authors:** Ashley C Griffin, Lu He, Anthony P Sunjaya, Andrew J King, Zubin Khan, Martin Nwadiugwu, Brian Douthit, Vignesh Subbian, Viet Nguyen, Mark Braunstein, Charles Jaffe, Titus Schleyer

**Affiliations:** Veterans Affairs Palo Alto Health Care System, Palo Alto, California, USA; Department of Health Policy, Stanford University School of Medicine, Stanford, California, USA; University of California, Irvine, Irvine, California, USA; The George Institute for Global Health, UNSW, Sydney, NSW, Australia; Department of Critical Care Medicine, University of Pittsburgh, Pittsburgh, Pennsylvania, USA; University of the Cumberlands, Williamsburg, Kentucky, USA; Division of Biomedical Informatics and Genomics, Tulane University School of Medicine, New Orleans, Louisiana, USA; Veterans Affairs Tennessee Valley Health Care System, Nashville, Tennessee, USA; Department of Biomedical Informatics, Vanderbilt University, Nashville, Tennessee, USA; University of Arizona, Tucson, Arizona, USA; Health Level Seven International, Ann Arbor, Michigan, USA; Georgia Institute of Technology School of Interactive Computing, Atlanta, Georgia, USA; Health Level Seven International, Ann Arbor, Michigan, USA; Regenstrief Institute Center for Biomedical Informatics, Indianapolis, Indiana, USA; Indiana University School of Medicine, Indianapolis, Indiana, USA

**Keywords:** fast healthcare interoperability resources, application programming interface, health information interoperability, medical informatics

## Abstract

**Objective:**

Understanding the current state of real-world Fast Healthcare Interoperability Resources (FHIR) applications (apps) will benefit biomedical research and clinical care and facilitate advancement of the standard. This study aimed to provide a preliminary assessment of these apps’ clinical, technical, and implementation characteristics.

**Materials and Methods:**

We searched public repositories for potentially eligible FHIR apps and surveyed app implementers and other stakeholders.

**Results:**

Of the 112 apps surveyed, most focused on clinical care (74) or research (45); were implemented across multiple sites (56); and used SMART-on-FHIR (55) and FHIR version R4 (69). Apps were primarily stand-alone web-based (67) or electronic health record (EHR)-embedded (51), although 49 were not listed in an EHR app gallery.

**Discussion:**

Though limited in scope, our results show FHIR apps encompass various domains and characteristics.

**Conclusion:**

As FHIR use expands, this study—one of the first to characterize FHIR apps at large—highlights the need for systematic, comprehensive methods to assess their characteristics.

## BACKGROUND

Since its creation in 2012, Health Level Seven International’s (HL7^®^) Fast Healthcare Interoperability Resources (FHIR^®^) interoperability standard for healthcare data exchange^1^ has gained enormous support worldwide, with FHIR applications (apps) being regularly developed and implemented across the healthcare landscape.^2^ In the United States, a patient-facing FHIR application programming interface (API) is now a federal requirement for electronic health record (EHR) certification as part of the Office of the National Coordinator for Health Information Technology final rule to implement the 21st Century Cures Act.^3^ The Centers for Medicare and Medicaid Service’s (CMS) Interoperability and Patient Access final rule will also start requiring payers to implement FHIR APIs for certain use cases in 2023.^4^ The European Union’s InteropEHRate and India’s Digital Health Blueprint efforts focus on implementing FHIR-based personal health records and data sharing with providers.^5^^,^^6^ New Zealand’s Ministry of Health and the United Kingdom’s National Health Service provide access to national patient identifier systems using FHIR APIs.^7^^,^^8^ These efforts, and many others, demonstrate that FHIR has emerged as the global interoperability standard for exchanging health data among systems.

As a result of increasing FHIR adoption and implementation of policies that prevent information blocking (eg, 21st Century Cures), patients are gaining greater access to their data. This has led to a growing number of tools that make it possible to aggregate health data from multiple providers and support patients to better organize, derive insights, and improve their health.^9–12^ For example, CMS’s Blue Button 2.0 FHIR API enables developers to create innovative tools for Medicare beneficiaries that facilitate access to medical claims data and connection to other tools and apps.^13^ Furthermore, FHIR allows third-party apps to connect to EHRs to facilitate clinician decision-making^14^ and supports querying and retrieving information from other clinical systems or health information exchanges.^15^^,^^16^ FHIR is also valuable for research communities to share data across institutions^17^ and aligns with FAIR (Findable, Accessible, Interoperable, Reusable) data principles. The National Institutes of Health has endorsed common data elements to promote scientific data reuse that provide support for FHIR and encourages use of FAIR principles in the revised data-sharing policy that will go into effect in 2023.^18^^,^^19^ Because FHIR enables scientific discovery and innovation in health information technologies, advancement of this standard supports opportunities to accelerate improvements in patient care and biomedical research.

Even with a few recent efforts to examine overall FHIR use in practice, the full extent to which FHIR apps are available throughout healthcare is not known. Jones et al^20^^,^^21^ conducted a survey of implementation of the SMART/HL7 Bulk FHIR Access API, which supports standardized population-level health data queries.^20^^,^^21^ Their findings revealed early progress in bulk data implementations among payers, EHR vendors, cloud vendors, and research or development organizations before the current United States Bulk FHIR Data Access regulations. Additionally, the Lantern Project, developed by MITRE Corporation, queries and displays publicly available FHIR API endpoints in the United States.^22^ In the study most similar to ours, Barker and Johnson^23^ developed an automated method to collect data from third-party apps connected to EHRs from 5 public galleries (Allscripts, Cerner Corporation, Epic Systems Corporation, Athenahealth, and SMART). Of the 734 apps they found, 112 apps in 2019 and 161 apps in 2021 described support for FHIR. However, while these websites and app galleries provide listings of FHIR apps, they are often EHR-specific, rely on manual entry, or do not capture the extent to which FHIR is being used. Having a better understanding of the range of real-world FHIR apps is critical to appreciate their breadth, inform development of novel app ecosystems, and promote advancement of the standard.

## OBJECTIVE

This study aimed to provide a preliminary overall assessment of real-world FHIR apps’ characteristics, including clinical domains and terminologies, technical specifications, and implementation details.

## MATERIALS AND METHODS

### Study design

This study was conducted in 3 phases: (1) identification and search of digital repositories and libraries with FHIR apps, (2) data extraction of app name, source, and contact information, and (3) collection of information about the apps via an electronic survey of app implementers and other stakeholders. The study was reviewed and considered exempt by the Indiana University Institutional Review Board (Protocol #12181).

### Eligibility criteria

We defined a FHIR app as a software application that uses FHIR as its interface to the data it requires and is designed for a human end user (patient, provider, or other individual) involved in patient care or related services. Inclusion criteria were apps that (1) reported using FHIR, (2) focused on healthcare (clinical, administrative, patients/caregivers, research, or educational applications), (3) had real-world users (including in pilot mode), and (4) had an English-language version available. Apps were excluded if they were in conceptual or planning phases.

### Search strategy

We electronically searched EHR App Gallery websites, public online repositories, AMIA/HL7 FHIR Applications Competitions, FHIR events, and publications in PubMed and Embase (Ovid) databases to identify potentially eligible FHIR apps. Search terms included variations of the following: *Fast Healthcare Interoperability Resources; application;* and *Application Programming Interface* (see Supplementary Appendix S1 for a full description of the search strategy). In addition to our wide-ranging search, we contacted individuals in our professional networks to assist with identification of apps. We extracted data from the repositories and compiled a list of potential FHIR apps with their sources and contact information (when available) in an Excel spreadsheet.

### Survey development and administration

We developed a Research Electronic Data Capture (REDCap^®^) electronic survey to collect from app implementers and other stakeholders the characteristics of FHIR apps, including developing organization, domain and target audience, FHIR specifications and terminologies, and implementation details (Supplementary Appendix S2). All survey questions were multiple choice and allowed multiple responses except for questions on development stage and FHIR release version. The survey was pilot-tested by FHIR implementers and informaticians prior to deployment. We then distributed the survey via email to the list of contacts. We used snowball sampling because this study was exploratory and the overall population of interest (all developers of real-world FHIR apps) could not be identified. Therefore, we encouraged survey recipients to forward the survey, share it on social media, or provide us with email addresses of additional stakeholders. The survey was also distributed on listservs (AMIA, HL7, HIMSS^®^, AeHIN), on social media, and to FHIR app developers among our professional contacts. All respondents were entered into a drawing for a free conference registration to the AMIA Annual Symposium or HL7 Connectathon. Survey data were collected between August 2021 and April 2022.

### Data synthesis and analysis

Survey responses were reviewed, and duplicate apps or apps not meeting inclusion criteria were removed. Descriptive statistics were used to summarize characteristics of the remaining apps.

## RESULTS

**Figure 1.**
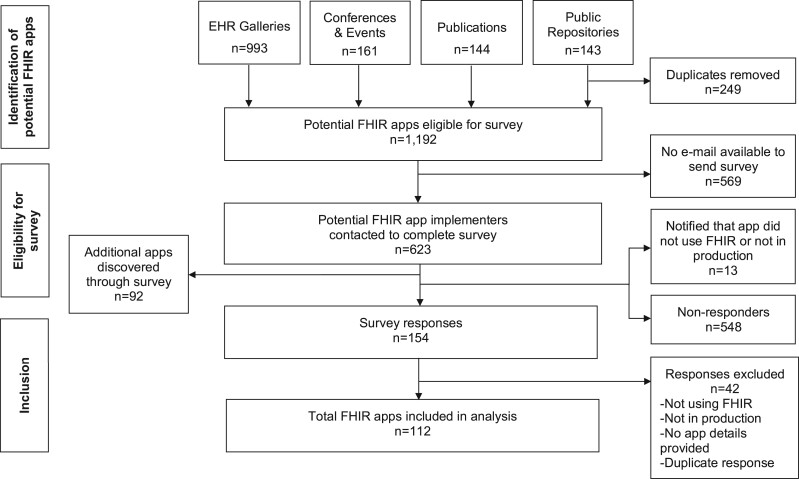
FHIR app identification, eligibility, and inclusion flowchart.

A total of 1192 potentially eligible FHIR apps were identified from the searches. Of the 623 individuals with a valid email whom we invited to complete the survey, we received 154 responses (25%). We excluded 42 responses that did not meet inclusion criteria, which resulted in information on 112 FHIR apps from 94 respondents (Figure 1).

Most respondents were from software vendors (*n* = 45) or academic institutions (*n* = 25) (Table 1). The apps primarily focused on clinical care (*n* = 74) or research (*n* = 45) and were targeted to health professionals (*n* = 75), patients (*n* = 47), or researchers (*n* = 37). Apps focused on a variety of health domains, with the most common being cardiovascular care (*n* = 28). Supplementary Appendix S3 shows apps’ characteristics by their intended purpose.

**Table 1.** Characteristics of survey respondents (*n* = 94) and FHIR apps for which information was collected (*n* = 112)

Respondents’ characteristics	Number
Organization	
Software vendor	45
Non-EHR vendor	40
EHR vendor	5
Academic institution	25
Health system	11
Payer	1
Other	12
Not specified	11
Role	
Management	43
Researcher	38
Developer	31
Sales/Marketing	11
Other	12
Not specified	12
Apps’ characteristics	Number
Purpose	
Clinical care	74
Research	45
Health information exchange	33
Public and population health	28
Education	18
Administration	15
Other	12
Target audience	
Health professionals	75
Patients	47
Researchers	37
Caregivers	31
Technical	30
Administrative/finance	16
Payers	15
Other	6
Health domain	
Cardiovascular	28
Diabetes	24
Critical care	22
Maternal and child	21
Cancer	21
Infectious disease	20
Mental health	20
None	10
Other	37
Not specified	29

*Note*: For all categories, multiple responses were possible. Respondents who selected “Other” had the option of entering free-text; see Supplementary Material for free-text responses in the full dataset. “Not specified” refers to items where the respondent did not choose any of the provided options and did not provide any free-text responses.

In terms of specifications, most of the apps used FHIR release R4 (*n* = 69), and nearly all used Patient (*n* = 96), Observation (*n* = 83), Condition (*n* = 78), or Medication (*n* = 71) resources (Table 2). Many respondents reported additional resources (*n* = 52) in free-text responses, such as Encounter, DocumentReference, and Practitioner resources (see Supplementary Material). For clinical terminologies, LOINC (*n* = 61) was most commonly reported, followed by SNOMED CT (*n* = 54), ICD-10 (*n* = 54), or RxNorm (*n* = 38). The majority used the Substitutable Medical Apps and Reusable Technologies (SMART)-on-FHIR platform (*n* = 55). However, many respondents did not select any of the listed API platforms (shown in table as “Not specified”) or selected “None.” This might be because their apps were developed with a proprietary API not listed in the survey, or the respondent might not have been familiar enough with the tools used to build the app.

**Table 2.** FHIR app specifications and implementation characteristics, *n* = 112

Specifications	Number
Version	
R 4	69
STU 3	13
DSTU 2	11
DSTU 1	4
Other	5
Not specified	12
Resources	
Patient	96
Observation	83
Condition	78
Medication	71
Procedure	58
Diagnostic report	53
Allergy intolerance	51
Other	52
Not specified	8
Clinical terminology standards	
LOINC	61
SNOMED CT	54
ICD-10	54
RxNorm	38
ICD-9	24
None	18
Other	15
Not specified	11
API platform	
SMART-on-FHIR	55
Apple HealthKit	12
Microsoft Azure	8
Google Cloud Healthcare	7
1UpHealth	7
Blue Button 2.0	7
None	16
Other	26
Not specified	31
CDS hooks	
No	74
Yes	25
Not specified	13
Implementation characteristics	Number
Year of implementation	
2021–2022	39
2018–2020	33
2015–2017	10
2012–2014	5
Other	2
Not specified	23
Stage	
Full use at multiple sites	56
Pilot study	31
Full use at development site	23
Not specified	2
Cost model	
Free	54
Cost per site	26
Cost per user	19
Other	27
Not specified	6
Type of app	
Web	67
EHR-embedded	51
Native Android	29
Native iOS	27
Other	14
Not specified	5
EHR gallery	
Epic App Orchard	32
Cerner App Gallery	18
Athenahealth Marketplace	9
Allscripts App Expo	6
None	49
Other	18
Not specified	13

*Note*: For all categories, multiple responses were possible except version and stage. Respondents who selected “Other” had the option of entering free-text; see Supplementary Material for free-text responses in the full dataset. “Not specified” refers to items where the respondent did not choose any of the provided options and did not provide any free-text responses.

These FHIR apps were implemented between 2011 and 2022, with nearly 35% (*n* = 39) deployed in 2021–2022. Most were implemented at multiple sites (*n* = 56) and were free to use (*n* = 54). The majority were stand-alone web-based (*n* = 67) or EHR-embedded (*n* = 51). Some were native Android (*n* = 29) or native iOS (*n* = 27) apps. Among the apps, 32 were available in the Epic App Orchard and 18 in the Cerner App Gallery, but 44% (*n* = 49) were not listed in any EHR gallery.

## DISCUSSION

### Principal findings

As one of the first efforts in characterizing real-world FHIR apps at large, this study identified a number of unique apps implemented throughout different healthcare settings. Most apps were developed by software vendors and used various API platforms, with the majority leveraging SMART-on-FHIR. SMART, developed by Boston Children’s Hospital Computational Health Informatics Program and the Harvard Medical School Department of Biomedical Informatics, is built on the FHIR API and resource definitions to create an open health app platform.^14^ The ability to connect third-party health apps to EHRs through SMART-on-FHIR has likely contributed to the growing number of real-world app implementations. We found that the majority of apps were being implemented across multiple health settings, which highlights the easily substitutable nature of FHIR. SMART Clinical Decision Support Hooks (CDS Hooks),^24^ which prompts decision support within a clinician’s workflow, was less commonly used (*n* = 25) despite most apps being targeted to health professionals. This may be due to the recent release of CDS Hooks in 2018.^25^ A small number of apps were focused on payers (*n* = 15), but this is expected to increase in the United States as payers will be required to implement FHIR APIs in 2023.^4^

Despite our multifaceted search strategy and survey distribution approach, the results were limited in terms of participation and number of responses. We found 1192 potentially eligible FHIR apps in our search of repositories. When we compared this list with the 161 apps identified by Barker and Johnson in 2020,^23^ 156 were on both lists. However, only 18 apps in our survey responses were on their list, which could be due to differences in time periods, apps no longer being in production, or limited survey participation. This highlights the need for more automated and comprehensive approaches to collect and maintain FHIR app characteristics. Expanding existing automated approaches^22^^,^^23^ to collect additional features of FHIR apps (domain, terminologies, implementation details, developing organizations) may be valuable in characterizing the use of FHIR. Overall, our exploratory study demonstrates the difficulty of identifying FHIR apps used in practice and their characteristics.

### Implications and opportunities for healthcare

Identifying real-world FHIR apps is challenging due to the increasing number of organizations developing apps and the heterogeneity of FHIR use across healthcare settings and countries. Our survey found that some apps were listed in various EHR galleries, but most were not listed in any gallery. This could be due to limited app maturity or the cost of listing apps in EHR galleries.^26–28^ This limitation makes it difficult for practitioners and organizations to discover apps to implement and identify opportunities for innovation that current apps do not fulfill. In addition, having a comprehensive repository of FHIR app use cases may be valuable to identify priority data elements for the United States Core for Data Interoperability (USCDI), which was adopted (version 1) as a standard in the 21st Century Cures Act final rule.^29^ The USCDI defines a common set of data classes and elements that health systems can capture and exchange. USCDI version 3, which was approved in July 2022, includes new data elements related to health equity, underserved populations, and emergency responses.^29^^,^^30^ It may be beneficial to query an existing registry for apps related to these priority areas. Notably, the Argonaut Project, which was initially tasked with accelerating FHIR use in 2014, has made foundational advancements in the FHIR standard and provides guidance to the USCDI.^31^ In addition, professional societies such as AMIA are important contributors to advancing the standard and national policies by fostering collaborations and providing educational events in the informatics community. Since 2018, the AMIA/HL7 FHIR Applications Competition has showcased more than 35 innovative FHIR apps. A continually updated FHIR app repository would support resource planning for FHIR-related competitions and “Connectathons” among the increasing number of global FHIR app developers and practitioners.

### Limitations

There were several limitations to this study. First, the population of global FHIR apps and their implementers is unknown, which limits our ability to generalize these findings. Second, our online searches likely did not retrieve all potentially eligible FHIR apps, and our survey distribution did not reach all FHIR implementers. The number of survey responses was low despite multiple distribution strategies, potentially resulting in sampling bias. Although it was not possible to determine how many FHIR developers the survey reached, we received 154 responses out of the 623 developers contacted (25%). While low response rates are common for voluntary online surveys, the technical nature of the questions or length of the survey (particularly for developers with multiple apps) may have contributed to low participation. Lastly, the FHIR standard is rapidly changing, and data from the survey were cross-sectional. Thus, the number and characteristics of FHIR apps may have changed outside of the period of data collection. These limitations prevent us from making conclusive statements about the broad FHIR landscape.

## CONCLUSION

The findings from this exploratory study demonstrate the momentum around FHIR and the diversity of apps currently implemented. Future work could leverage our publicly available survey dataset to examine app characteristics by target audience, clinical domain, or temporality or to assess the quality, reliability, or implementation complexities of real-world FHIR apps. Given the challenges in discovering real-world FHIR apps and their characteristics, this study highlights the importance of systematic data and metadata collection, monitoring, and maintenance of FHIR apps. Expanding existing repositories to encompass a more comprehensive global FHIR app registry would contribute to a better understanding of FHIR trends and support recent interoperability regulations. Such a repository would be valuable in fostering a vibrant community of FHIR implementers, researchers, and policymakers collectively supporting knowledge sharing, innovation, and progress of the standard worldwide.

## FUNDING

ACG and BD are currently supported by a VA Advanced Fellowship in Medical Informatics. The opinions expressed are those of the authors and not necessarily those of the Department of Veterans Affairs or those of the United States Government. APS is supported by a Scientia PhD scholarship from UNSW Sydney. AJK was supported in part by the National Library of Medicine and the National Heart, Lung, and Blood Institute under award numbers T15 LM007059 and R35 HL144804. VS was supported, in part, by the National Science Foundation under grant #1838745. TS acknowledges funding from the Lilly Endowment, Inc. Physician Scientist Initiative and the Indiana Clinical and Translational Sciences Institute, funded in part by grant #ULI TR002529 from the National Institutes of Health, National Center for Advancing Translational Sciences, Clinical and Translational Science Award.

## AUTHOR CONTRIBUTIONS

TS conceived the study concept and design. All authors contributed to the study design, search strategy, app identification, survey development, and data interpretation. All authors reviewed and approved the final manuscript.

## SUPPLEMENTARY MATERIAL

Supplementary material is available at *JAMIA Open* online.

## Supplementary Material

ooac077_Supplementary_Data

## Data Availability

Data are available in the Dryad Digital Repository: https://doi.org/10.5061/dryad.qrfj6q5k5.
