# Supplementary material for: Clinical, technical, and implementation characteristics of real-world health applications using FHIR
Source: JAMIA Open. 2022 Oct 12;5(4):ooac077. doi: 10.1093/jamiaopen/ooac077 (PMC9555876; doi:10.1093/jamiaopen/ooac077)
Supplement: ooac077_Supplementary_Data [file ooac077_supplementary_data.zip › Appendix2_Survey.pdf]

Appendix 2

A Global Survey on FHIR Applications 🔥

Are you developing software on Fast Healthcare Interoperability Resources (FHIR), the hottest standard around? If so, share your contributions by participating in a research study! Your participation is voluntary. The risk to you is minimal - we only ask for your name and email so we can track responses. The benefit? Help compile the most comprehensive catalog of FHIR applications worldwide! The survey will take ~10 min. to complete (~5 min. for each additional app).

Survey respondents will be entered into a drawing for a free registration to the AMIA Annual Symposium or HL7 Connectathon!

Please contact Titus Schleyer, DMD, PhD, at schleyer@regenstrief.org if you have any questions. Thanks!

PS: This study has been approved by the Indiana University Institutional Review Board (#12181).

General information

1) **What is your name?**
* must provide value

[ ]

2) **What is your email address?**
* must provide value

[ ]

3) **What is the name of your organization?**

[ ]

4) **Which of the following best describes your organization? (Check all that apply.)**

- ☐ Academic institution
- ☐ Health system
- ☐ Software vendor
- ☐ EHR vendor
- ☐ Payer
- ☐ Other [ ]

5) **What is your role? (Check all that apply.)**

- ☐ Developer
- ☐ Management
- ☐ Researcher
- ☐ Sales/marketing/corporate communication
- ☐ Other [ ]

6) **Are you using FHIR in any way in your software?**

This includes an application or implementation that uses FHIR to read and/or write data for healthcare purposes (e.g., clinical, educational, administrative, research, or other related care services).
* must provide value

- ○ Yes
- ○ No

reset

7) **Are you familiar with the AMIA/HL7 FHIR App Competition?**

We'd love to see your innovation at the competition!

- ○ Yes
- ○ No

reset

FHIR software specifications

8) **What is the name of your FHIR software?**
* must provide value

9) **Briefly describe how _____ is being used.**

Expand

10) **Select the most appropriate purpose of _____. (Check all that apply.)**
- Clinical care
- Public and population health
- Research
- Education
- Administration
- Health information exchange
- Other

11) **What stage of development is _____ in?**
- Pilot study
- Full use at development site
- Full use at multiple sites

12) **Briefly describe how the FHIR standard is implemented in _____ (e.g., user-facing software, machine-to-machine interface, etc.).**

Expand

13) **Which EHR galleries list _____? (Check all that apply.)**
- Epic App Orchard
- Cerner App Gallery
- Allscripts App Store
- Athena Marketplace
- None
- Other

14) **Which platforms does it support? (Check all that apply.)**
- EHR-Embedded
- Web
- Native iOS
- Native Android
- Other

15) **What is the software's cost model? (Check all that apply.)**
- Free
- Cost per user
- Cost per site
- Other

16) Does _____ support CDS Hooks?

- ○ Yes
- ○ No

17) Which FHIR release(s) does _____ use?

- ○ DSTU 1
- ○ DSTU 2
- ○ STU 3
- ○ R 4
- ○ Other ________

18) Which FHIR resources does _____ use? (Check all that apply.)

- ☐ AllergyIntolerance
- ☐ Condition
- ☐ DiagnosticReport
- ☐ Medication
- ☐ Observation
- ☐ Patient
- ☐ Procedure
- ☐ Other

19) Which FHIR API(s) does _____ use? (Check all that apply.)

- ☐ SMART on FHIR
- ☐ Apple HealthKit
- ☐ Google Cloud Healthcare
- ☐ 1UpHealth
- ☐ Microsoft Azure
- ☐ CMS Blue Button 2.0
- ☐ Other ________
- ☐ None

20) Which clinical terminology standards does _____ require? (Check all that apply.)

- ☐ LOINC
- ☐ ICD 9
- ☐ ICD 10
- ☐ SNOMED CT
- ☐ RxNorm
- ☐ None
- ☐ Other ________

21) Who is _____'s target audience? (Check all that apply.)

- ☐ Health professionals
- ☐ Patients
- ☐ Caregivers
- ☐ Researchers
- ☐ Admin/finance
- ☐ Payers
- ☐ Technical
- ☐ Other ________

22) **What is the approximate date of the implementation of _____ in a real-world setting? (i.e., used by health professionals, patients, or other users)**

Today D-M-Y

23) **What is the estimated number of current users?**

24) **Please share any links to the website or code repository of _____.**

25) **Please provide any additional information such as screenshots, videos, etc.**

Upload file

26) **Is there anything else regarding _____ that you would like to share?**

Expand

Thank you _____ for completing the survey!

27) **Please help us by sharing this survey.**

You can also optionally suggest names and email addresses of people or organizations for us to invite.

Expand

28) **Click here to opt out of having identifiable information from this survey mentioned in any presentations or publications.**

☐ Opt out

29) **Click here to opt out of having identifiable information from this survey shared in a public online repository.**

☐ Opt out
