# Supplementary material for: Clinical, technical, and implementation characteristics of real-world health applications using FHIR
Source: JAMIA Open. 2022 Oct 12;5(4):ooac077. doi: 10.1093/jamiaopen/ooac077 (PMC9555876; doi:10.1093/jamiaopen/ooac077)
Supplement: ooac077_Supplementary_Data [file ooac077_supplementary_data.zip › Appendix3_Characteristics_by_Purpose_Visualization.docx]

**Appendix 3. FHIR App Characteristics Color-Coded by App Purpose (n=112)**


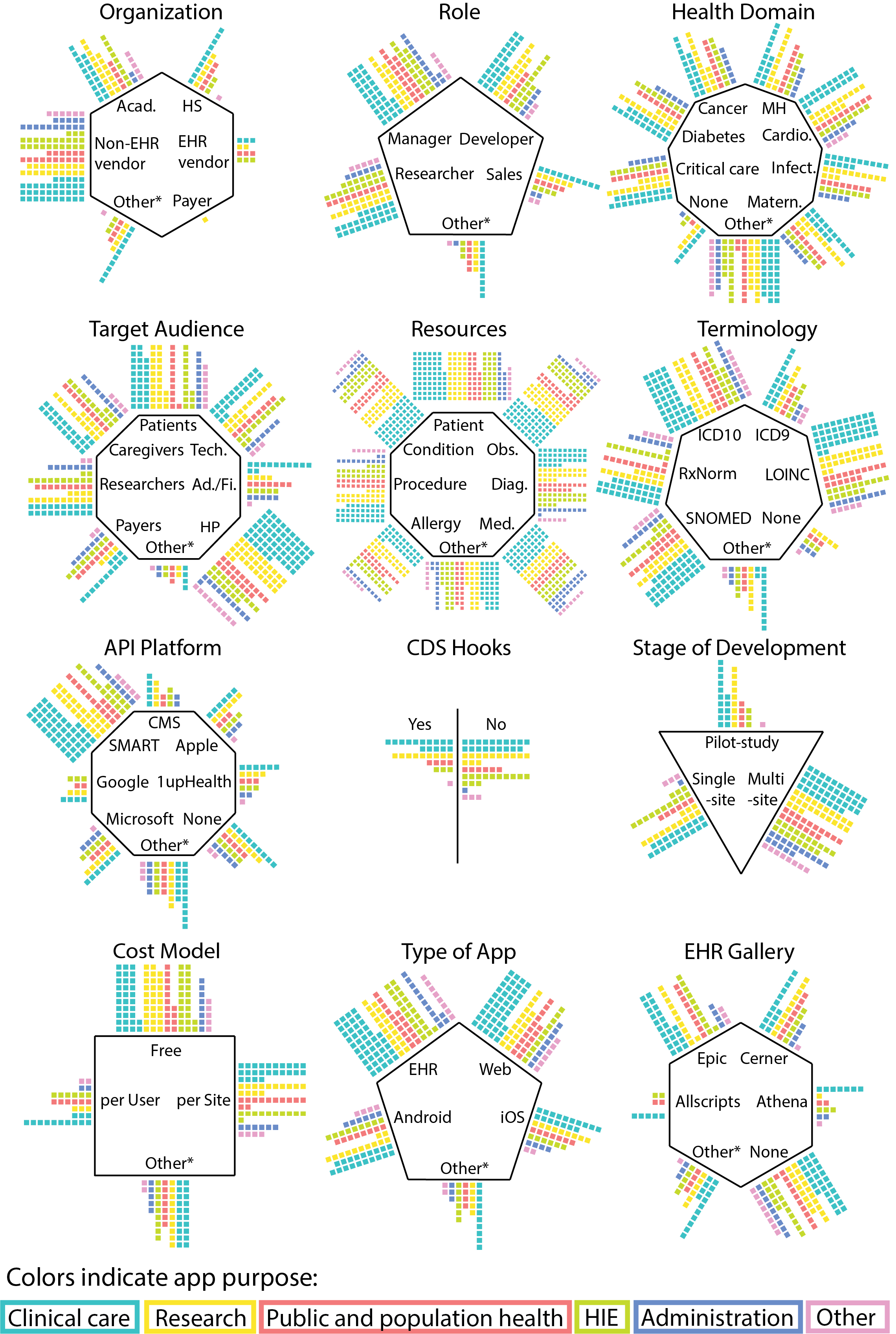


Each square indicates one response; however, apps appear more than once if they were intended for more than one purpose. *See Supplementary Materials for the free-text responses in the full dataset.

Abbreviations are as follows: Acad.=Academic institution; Ad./Fi.=Administrative and financial; Allergy=AllergyIntolerance; API= Application Programming Interface; Cardio.=Cardiovascular; CMS=Centers for Medicare and Medicaid Services Blue Button 2.0; Diag.=DiagnosticReport; EHR=Electronic Health Record; HIE=Health Information Exchange; HP=Health professionals; HS=Health system; Infect.=Infectious disease; Matern.=Maternal and child care; Med.=Medication; MH=Mental health; Obs.=Observation; Tech.=Technical
