# Supplementary material for: Clinical, technical, and implementation characteristics of real-world health applications using FHIR
Source: JAMIA Open. 2022 Oct 12;5(4):ooac077. doi: 10.1093/jamiaopen/ooac077 (PMC9555876; doi:10.1093/jamiaopen/ooac077)
Supplement: ooac077_Supplementary_Data [file ooac077_supplementary_data.zip › Appendix1_Search_Strategy.docx]

**Appendix 1. FHIR App Search Strategy**

Potential FHIR apps were searched in EHR galleries, conferences and events, publications, and public repositories. For each category, search terms included combinations of *Fast Healthcare Interoperability Resources; FHIR; application; app; Application Programming Interface;* and *API.* For publications, search terms could appear anywhere within the title, abstract, or keywords. All searches were conducted prior to July 1, 2021.

| **Category** | **Source** |
| --- | --- |
| EHR Galleries | Epic App Orchard: <https://apporchard.epic.com/>  Cerner App Gallery: <https://code.cerner.com/apps>  Allscripts App Expo: <https://expo.allscripts.com/Developers>  Athenahealth Marketplace: <https://marketplace.athenahealth.com/> |
| Conferences & Events | HL7 FHIR Applications Roundtable (2016-2018): <http://www.hl7.org/events/fhirapps.cfm>  AMIA/HL7 FHIR Applications Competitions (2018-2020): <https://amia.org/amia2021/fhir-app-competition> |
| Publications | PubMed: <https://pubmed.ncbi.nlm.nih.gov/>  Embase (Ovid): <https://www.wolterskluwer.com/en/solutions/ovid/embase-903>  Search Strings: "FHIR" OR "Fast Healthcare Interoperability Resources" OR “FHIR App*” OR “FHIR Application*” |
| Public Repositories | HL7 FHIR Application Registry: <http://fhir.org/implementations/registry/>  SMART App Gallery: <https://apps.smarthealthit.org/apps>  1upHealth FHIR App Gallery: <https://1up.health/health-apps> |
